# Visual Turing test is not sufficient to evaluate the performance of medical generative models

**DOI:** 10.1186/s41747-023-00347-8

**Published:** 2023-07-10

**Authors:** Shoichiro Yamamoto, Akinori Higaki

**Affiliations:** Department of Cardiology, Pulmonology, Hypertension and Nephrology, Ehime University Graduate School of Medicine, 454 Shitsukawa, Toon, Ehime 791-0295 Japan

**Keywords:** Artificial intelligence, Generative adversarial networks, Image processing (computer-assisted), Medical image synthesis, Visual Turing test

To the Editor,

We read with great interest the article by Wang et al. [1], reporting that generative adversarial networks (GANs) could generate synthetic ground glass opacities (GGOs) in computed tomography. While we appreciate their ambitious research to advance clinical radiology, we feel that the performance evaluation of the GANs is insufficient for their aim.

In their study, the authors stated that the model performance was evaluated by both subjective and objective approaches, namely the visual Turing test (VTT) and the distribution of radiomic features. We agree that VTT is a suitable approach to assess the realism of synthesized medical images [2], but a low VTT score does not guarantee the diversity of the generated data; it tells us they just look real. As the authors admitted as a limitation in the “Discussion” section, about 40% of the distributions of the radiomic features (e.g., NGTDM coarseness) were significantly different between generated and original images. Therefore, we suspect that their generative model may only be able to produce biased images due to the so-called mode collapse phenomenon [3]. If this were the case, it would diminish the usefulness of the data augmentation for classification tasks.

It is true that there is no single universal metric to assess the model performance and the quality of generated data; therefore, we need to combine several indicators, such as inception score, Fréchet inception distance, and geometry score [4, 5]. In addition to these, the image quality can be also evaluated quantitatively by NIQE, PIQE, and BRISQUE scores, as Oyelade and colleagues have demonstrated for mammography images [6]. As a practical matter, the images presented in the article are so small in size and resolution that the readers cannot fully appreciate what kind of images the GAN model has produced.

In summary, we believe that the authors need to provide more example images of the generated GGO and evaluate their GAN in several other ways to ensure the quality of data synthesis.

## Data Availability

Not applicable.
